# The relationship between physical exercise and school adaptation of junior students: A chain mediating model

**DOI:** 10.3389/fpsyg.2022.977663

**Published:** 2022-09-14

**Authors:** Meng-Zhu Bai, Shu-Jun Yao, Qi-Shuai Ma, Xun-Ling Wang, Chao Liu, Ke-Lei Guo

**Affiliations:** ^1^School of Physical Education, Huaibei Normal University, Huaibei, China; ^2^Department of Physical Education, Guang Dong Technology College, Zhaoqing, China; ^3^School of Physical Education and Health, Zhaoqing University, Zhaoqing, China

**Keywords:** physical exercise, psychological resilience, sports learning motivation, school adaptation, chain mediation

## Abstract

**Objective:**

This study explores the relationship between physical exercise and school adaptation of junior middle school students and constructs a chain intermediary model through the intermediary role of psychological resilience and sports learning motivation.

**Methods:**

Using the stratified cluster sampling method, 930 junior middle school students in Anhui Province were measured in group psychology by using the physical exercise rating scale, adolescent psychological resilience scale, physical learning motivation scale and school adaptation scale. The statistical software SPSS 23.0 and process plug-in were used for statistical processing, and the common method deviation test was carried out by Harman single-factor control method. Finally, the bootstrap sampling test method and process plug-in were used to test the significance of intermediary effect.

**Results:**

(1) The direct prediction effect of physical exercise on school adaptation is remarkable (*t* = 4.60, *p* < 0.01); (2) psychological resilience and sports learning motivation are the intermediary variables of the relationship between physical exercise and school adaptation; (3) psychological resilience and sports learning motivation play a chain mediation role in the relationship between physical exercise and school adaptation. The intermediary effect is composed of three indirect effects: physical exercise → psychological resilience → school adaptation (95% Cl: 0.004, 0.041), physical exercise → physical learning motivation → school adaptation (95% Cl:0.019, 0.065), physical exercise → psychological resilience → physical learning motivation → school adaptation (95% Cl:0.002, 0.021).

**Conclusions:**

Physical exercise can directly improve the school adaptation of junior middle school students, which can also affect junior middle school students’ school adaptation indirectly through psychological resilience or sports learning motivation, and it can influence school adaptation through the chain mediation of psychological resilience and sports learning motivation.

## Introduction

School adaptation includes students' performance in school, their emotional attitude toward school life and their activity in school activities (Zhang et al., 2021). It is a process of interaction between students, school environment and school activities (Kang et al., 2021). School adaptation of middle school students not only directly affects their learning state and achievement in school, but also affects the growth of their personality, the formation of their values and the development of their social adaptability (Guo et al., 2021). The research shows that parent–child, teacher–student, peer relationship is positively correlated with junior high school students’ school adaptation, and there is also a significant positive correlation between middle school students’ mental resilience and school adaptation (Ma, 2017). It is not difficult to find that the influence of school adaptation on junior high school students is not only reflected in academic, interpersonal and mental health aspects, but also can predict their social adaptation ability. Therefore, it is very important to find ways to promote junior high school students’ school adaptation, so as to further promote the all-round development of teenagers. A large number of studies have shown that physical exercise can help students adapt to the school environment, increase the ability of unity and cooperation, and promote students’ growth and physical and mental health development (Yan et al., 2020). Previous studies have found that good physical exercise can promote academic performance and bring academic improvement to elementary and middle school students (Li et al., 2021). According to the 13th 5-year plan for the Development of China’s National Education, it is necessary to comprehensively enhance students’ physique, improve their volitional quality and mental health, strengthen school physical education work, and cultivate a healthy and strong-willed generation of new students (Xi, 2013). The Decision of the Central Committee of the Communist Party of China on Some Major Issues of Comprehensively Deepening the Reform also emphasizes that physical education courses and physical exercises should be strengthened to promote the cultivation of students’ physical and mental health behaviors. Some scholars have found that physical exercise not only promotes the healthy development of students, but also plays an important role in cultivating students’ core qualities (Otundo and Garn, 2019). Therefore, hypothesis 1 is proposed in this study: junior high school students’ physical exercise can positively predict school adaptation.

One of the mediating effects of this study is the mediating effect of psychological resilience. Psychological resilience is reflected in the ability of individuals to adapt well to external pressure (Xi and Sang, 2002; Gao, 2019). Junior high school students are in the critical period of psychological development, so they need a good level of psychological resilience. Some scholars believe that physical exercise is helpful to cultivate and improve the psychological resilience of middle school students, and experimental studies have shown that different degrees of physical exercise can improve the psychological resilience of students to different degrees (Hu, 2019). A large number of studies have shown that psychological resilience is a positive and good psychological quality, and is closely related to individual adaptation, and can even be said to be the most important determinant of individual adaptation (Wang, 2022). Students with higher level of psychological resilience can show better adaptability in the face of pressure (Zhang, 2015). Therefore, it can be concluded that the relationship between psychological resilience and school adaptation is positively correlated with significant effect, which is consistent with the research results of Zhang et al. (2017) and Guo et al. (2012). In conclusion, hypothesis 2 is proposed in this study: psychological resilience plays a mediating role between physical exercise and school adaptation of junior high school students.

Another focus of this study on the mediating effect is the mediating effect of sports learning motivation. Learning motivation can stimulate and guide students to study hard, directly promote students’ interest in learning and learning results, and is an important factor affecting students’ course adaptation (Li and Liu, 2010). Wei (2015) believes that learning motivation is a driving force to promote students’ learning activities and improve their learning efficiency. Psychologists point out that students’ learning motivation is mainly achievement motivation, and many scholars have concluded that there is a significant positive correlation between achievement motivation of junior high school students and school adaptation through investigation and research (Paul et al., 2012). As an internal driving force, sports learning motivation guides students’ sports learning, which plays an important role in students’ sports learning effect, physical exercise and cultural learning (Liu, 2020). Some scholars believe that sports learning motivation can lead students to actively participate in sports, and strengthening physical exercise can promote students’ sports learning motivation (Yu-Jy et al., 2020). In this way, a virtuous cycle of physical exercise and learning motivation is formed. Physical activity plays a positive role in promoting students’ learning motivation and academic performance (Liu and Lipowski, 2021). More research shows that there is a significant positive correlation between learning motivation and academic performance (Qi et al., 2013; Huang, 2015). Therefore, learning motivation can positively predict students’ learning behavior. In conclusion, hypothesis 3 is proposed in this study: sports learning motivation plays a mediating role between physical exercise and school adaptation of junior high school students.

Peng (2013) pointed out that both learning motivation and psychological resilience can predict high school students' English performance to a certain extent. The research of Zhao and Chen (2018), Brandon et al. (2018), and Zhao (2021) showed that psychological resilience is significantly positively correlated with learning motivation. Through correlation analysis, Niu et al. (2012) showed that there was a significant correlation between psychological resilience and all dimensions of learning motivation. Zeng and Liu (2013) pointed out that there was a significant positive correlation between sports motivation and psychological resilience scores of athletes in various dimensions. In conclusion, learning motivation and psychological resilience are closely related. Therefore, this study hypothesizes that learning motivation and psychological resilience, as internal and external factors, can jointly influence school adaptation and have a potential dual mediating role. Therefore, this study proposed hypothesis 4: psychological resilience and motivation of sports learning play a chain mediating role between physical exercise and school adaptation.

**Figure 1:**
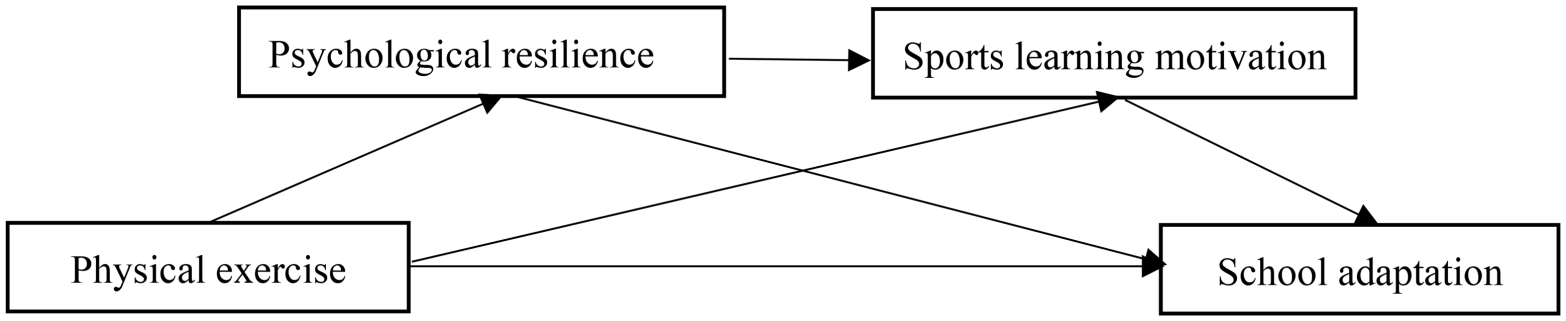
Conceptual framework.

To sum up, in order to investigate the internal mechanism between physical exercise and school adaptation, this study plans to build a chain mediating model (as shown in Figure 1), and will verify the following aspects: (1) Physical exercise significantly positively predicts school adaptation of junior high school students; (2) Psychological resilience plays an independent intermediary role between physical exercise and school adaptation; (3) Sports learning motivation plays an independent intermediary role between physical exercise and school adaptation; (4) Psychological resilience and sports learning motivation play a chain intermediary role between physical exercise and school adaptation.

## The research methods

### The participants

We adopt stratified cluster sampling method. According to the characteristics of administrative division the geographical location and economic level, we select three regions covering urban and rural areas from Southern Anhui, middle Anhui, and north Anhui, a province of China. We focus on six junior high schools, and from each grade of each junior high school we randomly select two classes as participants. The survey informed consent was send prior to the survey, and all participants were required to fill the consent forms. A total of 1,021 people completed the questionnaire. After eliminating invalid questionnaires caused by fixed answers and lack of data, 930 valid questionnaires were collected, with an effective rate of 91.09% (502 in urban areas and 428 in rural areas, 527 males and 403 females; 293 in junior one, 262 in junior two, 375 in junior three. The average age of the subjects was 13.71 ± 0.891 years.).

The test was conducted with the consent of the head teacher, parents, and the subjects themselves. The experimenters are college students majoring in sports psychology. The collective test was adopted, emphasizing the principles of anonymous filling, data confidentiality, and voluntary filling. Being in accordance with the Declaration of Helsinki, the research design has passed the ethical review procedure of the human research ethics committee of Huaibei Normal University. In this process, all invited participants are voluntary, thus confidentiality has been guaranteed, and written informed consent of all participants’ parents or guardians has been obtained.

### Research tools

#### Physical activity rating scale

We adopt the physical exercise grade scale revised by Liang (1994). This scale, includes three entries from physical exercise intensity, time and frequency of physical exercise capacity. The participation of physical exercise are measured. Every entry has been subdivided into five levels. Ranging from 1 to 5 points. The total amount of physical exercise score = exercise intensity score × (exercise time scoring–1) × exercise frequency score. The higher the score, the higher the amount of physical exercise. A score of 19 or less was classified as low exercise, 20–42 as moderate exercise, and 43 or greater as high exercise. According to the previous experience, this study divided the small amount of physical exercise into two parts: no physical exercise and small amount of exercise. No physical exercise is equal to or less than 4 points, light exercise 5–19 points. Therefore, the physical exercise in this study was divided into four levels, from “1 = no physical exercise” to “4 = large amount of physical exercise” (Xia et al., 2018). The scale has high reliability and validity, and its retest reliability *r* = 0.82. In this study, the Cronbach’s *α* of the scale was 0.79.

#### Adolescent resilience scale

Psychological resilience was measured using the Adolescent Mental Resilience Scale developed by Hu and Gan (2008). There are 27 questions on five dimensions: goal focus, emotional control, positive cognition, family support, and interpersonal assistance (e.g., “Failure always makes me feel discouraged.”), Cronbach’s *α* is greater than 0.7. Likert 5-point evaluation was adopted in the questionnaire, ranging from “1 = Completely Not Conforming” to “5 = Completely Conforming.” Among them, the reverse scoring questions of 1, 2, 5, 6, 9, 12, 15, 16, 17, 21, 26, and 27 were rated as “1 = Completely Conforming” to “5 = Completely Not Conforming.” The higher the score was, the higher the level of mental resilience of adolescents. In addition, a previous study showed that the scale worked well in a sample of junior high school students (Xie et al., 2014). Fit index of confirmatory factor analysis in this study: *χ*^2^/DF = 2.50, RMSEA = 0.07, CFI = 0.95, GFI = 0.81, AGFI = 0.91, NNFI = 0.92, and NFI = 0.91.The goodness of fit is significantly better, indicating that the scale has good structural validity (Table 1).

**Table 1:** Results of confirmatory factor analysis.

Factor naming	*χ*^2^/df	CFI	NFI	GFI	NNFI	RMESA
Psychological resilience	2.50	0.95	0.91	0.81	0.92	0.07
Sports learning motivation	3.55	0.99	0.94	0.92	0.91	0.08
School adaptation	4.05	0.93	0.95	0.90	0.97	0.05

#### Physical education learning motivation scale

The sports learning motivation scale is used to measure the sports learning motivation. The scale is a sports situational motivation scale compiled by Guay and Vallerand (1996) and others to investigate the sports learning motivation of junior middle school students. It was revised by the Chinese scholar Su (2007) and established with the theme of “why do you engage in this sport” to conduct a targeted questionnaire survey on teenagers. The results show that the scale has been well applied in the sample of junior high school students (Guo, 2012). The scale consists of four dimensions of intrinsic motivation, external regulation, discriminating principle and lack of motivation, with a total of 27 questions (e.g., “I find PE class interesting.”). Likert's 5-point evaluation was adopted in the questionnaire, ranging from “1 = completely disagrees” to “5 = completely agrees.” When the scores of questions 1, 5, 9, and 13 were added together, the lower the value was, the lower the internal motivation was, and the higher the score was, and vice versa. When the scores of questions 2, 6, 10, and 14 are added up, the lower the value, the weaker the discrimination principle; When the scores of questions 3, 7, and 11 are added up, the lower the value, the weaker the externalization principle. Add up the scores on questions 4, 8, and 12. The lower the number, the lower the lack of motivation. Cronbach’s *α* of the four subscales were 0.79, 0.75, 0.81, and 0.60, respectively. The confirmatory factor analysis fitting index of the scale in this study: CFI = 0.99, GFI = 0.92, TLI = 0.98, RMSEA = 0.08, *χ*^2^/DF = 3.55, NFI = 0.94, and NNFI = 0.91. The goodness of fit is significantly better, indicating that the scale has good structural validity (Table 1).

#### School adjustment scale for junior high school students

School adaptation scale for junior middle school students compiled by Cui (2008) was used to measure school adaptation. The study proves that this scale is widely applicable to the measurement of school adaptation of junior high school students in China (Chen et al., 2019). A total of 27 questions are included in five dimensions, including routine adaptation, homework adaptation, peer relationship, teacher–student relationship, and school attitude, (e.g. “I am often absent-minded when studying”), using Likert 5-point evaluation, from “1 = completely disagrees” to “5 = completely agrees,” except for questions 3, 5, 14, 16, and 23, all the others are reverse scoring, from “1 = completely agrees” to “5 = completely disagrees,” the higher the score is, the better the school adaptation status. Cronbach’s *α* of the total questionnaire was 0.78. The confirmatory factor analysis fitting index of the scale in this study: *χ*^2^/DF = 4.05, CFI = 0.93, GFI = 0.90, TLI = 0.92, RMSEA = 0.05, NFI = 0.95, and NNFI = 0.97. The goodness of fit is significantly better, indicating that the scale has good structural validity (Table 1).

### Statistical analysis

This study has adopted IBM SPSS23.0 and AMOS26.0 statistical software for all data analyses. After the questionnaires were collected, all the data have been processed as follows: (1) Exploratory factor analysis was performed on all scales by SPSS23.0; (2) confirmatory factor analysis was performed on all scales by AMOS26.0; (3) internal consistency was tested for all scales by SPSS23.0; (4) the Harman single-factor method has been adopted for the common method deviation test; (5) Pearson correlation analysis has been applied to calculate the relationship among physical exercise, psychological resilience, sports learning motivation, school adaptation and continuous variables of normal distribution are expressed as mean (M) ± standard deviation (SD); (6) By using the SPSS macro program compiled by Hayes in SPSS23.0, the mediating effects of psychological resilience and sports learning motivation on the relationship between physical exercise and school adaptation, and the chain mediating effects of psychological resilience and sports learning motivation on the relationship between physical exercise and school adaptation were verified, respectively; and (7) Model 6 in the SPSS macro program compiled by Hayes has been adopted for the chain mediating test. In this study, the significance level is set as *p* < 0.05.

## Results

### Common method deviation control and testing

Common method deviation refers to the error caused by the same measured environment, the same data source or the grader. Since the data in this study came from the questionnaire survey, common method deviation may occur. There are many methods to test common method deviation, and Harman single-factor test is the most commonly used method in this study. The results show that the characteristic roots of the five factors are greater than 1, and the first factor can explain 36.221%, less than 40% of the standard threshold value, indicating that there is no serious common method bias problem in this study.

### Describe statistical and correlation analyses

As shown in Table 2, Pearson bivariate correlation analysis results among all variables show that physical exercise is significantly positively correlated with school adaptation, sports learning motivation, and psychological resilience; sports learning motivation and psychological resilience are significantly positively correlated with school adaptation; and sports learning motivation and psychological resilience are significantly positively correlated with school adaptation. Hypothesis 1 is verified.

**Table 2:** Average, standard deviation, and correlation coefficient of each variable.

	*M*	SD	1	2	3	4
1. Physical exercise	3.047	0.892	1			
2. Psychological resilience	3.121	0.402	0.086^**^	1		
3. Sports learning motivation	3.442	0.699	0.138^**^	0.349^**^	1	
4. School adaptation	3.825	0.738	0.149^**^	0.386^**^	0.469^**^	1

** At the level of 0.01, the correlation is significant.

According to Wen and Ye (2014) ‘s suggestions on the intermediary effect test, regression analysis is carried out on the chain intermediary effect model, and the analysis results are shown in Table 3. According to the data results in Table 3, physical exercise can significantly and positively predict the school adaptation of junior middle school students. The total effect is 0.182 (*p* < 0.01), and the direct effect is 0.114 (*p* < 0.01). Therefore, hypothesis 1 is true. After incorporating psychological resilience and sports learning motivation into the regression equation, physical exercise significantly positively predicted psychological resilience (*β* = 0.083, *p* < 0.01), and sports learning motivation (*β* = 0.073, *p* < 0.01). Psychological resilience significantly positively predicts school adaptation (*β* = 0.068, *p* < 0.01). And sports learning motivation (*β* = 0.359, *p* < 0.01). Sports learning motivation significantly positively predicts school adaptation (*β* = 1.402, *p* < 0.01). At this time, physical exercise can still significantly predict school adaptation (*β* = 0.114, *p* < 0.01). It can be concluded that psychological resilience and sports learning motivation play a part of intermediary role between physical exercise and school adaptation. Assumptions 1 and 3 are supported by data.

**Table 3:** Analysis of regression relationship of variables.

Effect	Item	Effect	SE	*t*	*p*	LLCI	ULCI
Direct effects	Physical exercise ⇒ school adaptation	0.114	0.053	2.149	<0.01	0.034	0.066
Indirect effect process	Physical exercise ⇒ psychological resilience	0.083	0.04	2.056	<0.01	0.164	0.253
	Physical exercise ⇒ sports learning motivation	0.073	0.021	3.485	<0.01	0.138	0.171
	Psychological resilience ⇒ school adaptation	0.068	0.072	9.482	<0.01	0.036	0.068
	Psychological resilience ⇒ sports learning motivation	0.359	0.026	13.771	<0.01	0.281	0.327
	Sports learning motivation ⇒ school adaptation	1.402	0.123	11.413	<0.01	0.105	0.241
Total effect	Physical exercise ⇒ school adaptation	0.182	0.014	15.335	<0.01	0.217	0.189

### An analysis of the mediating effect of psychological resilience between physical exercise and school adaptation

The mediating effect between physical training and school adaptation was analyzed by using the sequential test method in the process of the mediating effect test (step-by-step test method) proposed by Wen and Ye (2014).

As can be seen from Table 4, physical exercise has a significant direct prediction effect on school adaptation (*t* = 4.60, *p* < 0.01), and physical exercise has a significant direct prediction effect on psychological resilience (*t* = 2.626, *p* < 0.01). When both physical exercise and psychological resilience were added into the regression equation, the prediction effect of physical exercise on school adaptation was still significant (*t* = 3.878, *p* < 0.01), and psychological resilience was also significantly positive in predicting school adaptation (*t* = 12.475, *p* < 0.01). According to the formula *a* × *b*/*c* for calculating the effect proportion, psychological resilience played a partial mediating role between physical training and school adaptation, accounting for 21.909% of the effect. Hypothesis 2 is verified.

**Table 4:** Analysis results of the mediating effect of psychological resilience.

	School adaptation			Psychological resilience			School adaptation		
	*B*	SE	*t*	*B*	SE	*t*	*B*	SE	*t*
Constant	3.449^**^	0.085	40.47	3.004^**^	0.047	64.253	1.373^**^	0.184	7.454
Physical exercise	0.123^**^	0.027	4.60	0.039^**^	0.015	2.626	0.097^**^	0.025	3.878
Psychological resilience							0.691^**^	0.055	12.475
*R* ^2^	0.022			0.007			0.163		
Adjust the *R*^2^	0.021			0.006			0.161		
*F*	*F* (1,928) = 21.156, *p* = 0.000			*F* (1,928) = 6.893, *p* = 0.009			*F* (2,927) = 90.150, *p* = 0.000		

**p < 0.01.

### Analysis of the mediating effect of physical education learning motivation on physical exercise and school adaptation

As can be seen from Table 5, physical exercise has a significant direct prediction effect on school adaptation (*t* = 4.60, *p* < 0.01), and physical exercise has a significant direct prediction effect on sports learning motivation (*t* = 4.253, *p* < 0.01). When both physical exercise and sports learning motivation were added into the regression equation, the prediction effect of physical exercise on school adaptation was still significant (*t* = 2.953, *p* < 0.01), and sports learning motivation could also significantly positively predict school adaptation (*t* = 15.674, *p* < 0.01). Sports learning motivation played a partial intermediary role between physical exercise and school adaptation, the effect accounted for 42.322%. Hypothesis 3 is verified.

**Table 5:** Analysis results of the mediating effect of sports learning motivation.

	School adaptation			Sports learning motivation			School adaptation		
	*B*	SE	*t*	*B*	SE	*t*	*B*	SE	*t*
Constant	3.449**	0.085	40.47	3.112**	0.081	38.481	1.948**	0.122	15.948
Physical exercise	0.123**	0.027	4.60	0.108**	0.025	4.253	0.071**	0.024	2.953
Sports learning motivation							0.482**	0.031	15.674
*R* ^2^	0.022			0.019			0.227		
Adjust the *R*^2^	0.021			0.018			0.225		
*F*	*F* (1,928) = 21.156, *p* = 0.000			*F* (1,928) = 18.092, *p* = 0.000			*F* (2,927) = 136.203, *p* = 0.000		

**p < 0.01.

### The chain mediating effect of psychological resilience and sports learning motivation on physical exercise and school adaptation

The Bootstrap method of deviation correction was used to test the chain mediating effect of psychological resilience and motivation of physical education learning on physical exercise and school adaptation (Fang and Zhang, 2013). Five-thousand Bootstrap samples were randomly selected from the initial sample to estimate the indirect effect.

**Figure 2:**
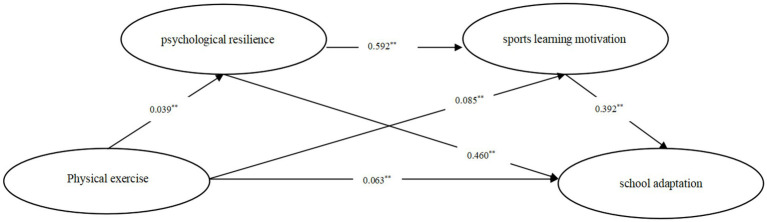
Chain mediation model of physical exercise and school adaptation. **p < 0.01.

Table 6 shows the 95% confidence intervals of the Bootstrap sampling test for each path. If the number 0 is not included, the mediating effect is significant. According to Table 6, the 95% confidence intervals of the three influence paths did not contain the number 0, indicating that psychological resilience had a significant mediating effect (the mediating effect value was 0.018), and the effect size was 14.634% (95%CI: 0.004, 0.041). Sports learning motivation has significant mediating effect (mediating effect value is 0.033), effect size is 26.829%, (95%CI, 0.019, 0.065); Psychological resilience had a significant chain mediating effect on sports learning motivation (the mediating effect value was 0.009), and the effect size was 7.317% (95%CI, 0.002, 0.021). The model diagram is shown in Figure 2. Hypothesis 4 is verified.

**Table 6:** Bootstrap analysis of mediation effect significance test.

Affect the path	Effect	Boot SE	Boot LLCI	Boot ULCI
Physical exercise ⇒ psychological resilience ⇒ school adaptation	0.018	0.01	0.004	0.041
Physical exercise ⇒ sports learning motivation ⇒ school adaptation	0.033	0.012	0.019	0.065
Physical exercise ⇒ psychological resilience ⇒ sports learning motivation ⇒ school adaptation	0.009	0.005	0.002	0.021

## Discussion

### The relationship between physical exercise and school adaptation

The results of this study show that there is a significant positive correlation between physical exercise and school adaptation of junior high school students in Anhui Province (*p* < 0.01), and the predictive effect is still significant after the inclusion of mediating variables (*p* < 0.01), which is consistent with the research results of Yan et al. (2020). Physical exercise can temper the will of the people, to help students cultivate good living habits and personality quality (Li, 2020), studies have shown that ability to adapt to the campus sports group is higher than in mature group (Zhang et al., 2017), enhance the students’ school adjustment can help middle school students in the school to build good partner relationship, promote the formation of the young people personality development, the values at the same time, Laying a foundation for lifelong development(Suhlmann et al., 2018). According to physical education theory, exercisers with strong management of exercise motivation can participate in exercise more actively, and scientific physical exercise methods can promote students’ physical and mental health and adapt to the school environment (Yu and Yang, 2009; Guo, 2019). Physical exercise cultivates students’ sense of cooperation, collective sense of honor, sense of responsibility, and organizational discipline. In this process, students can improve their personality, temper their wills, promote the formation of healthy behaviors and develop their core qualities (Nigg et al., 2011).

### The mediating role of psychological resilience and sports learning motivation

In this study, there is a significant positive correlation between psychological resilience and school adaptation, which is consistent with the research results of Long (2017) and Zhang (2015), further confirming the mediating role of psychological resilience between physical exercise and school adaptation of junior high school students. It shows that improving the psychological resilience of junior high school students is the key way for physical exercise to influence their school adaptation (Xie et al., 2016). Physical exercise can regulate the attention of junior high school students, improve their cognitive function, improve their emotional management level. The general population model proposes that the higher the level of resilience shown in response to adversity in life, the stronger the performance in the three main development areas of ability development: academic performance, discipline compliance or antisocial behavior, and peer communication ability (Joseph, 1996). According to the research on psychological resilience and school adaptation, there is a significant positive correlation between them. The higher the school adaptation ability of students with high psychological resilience is, the more conducive to their successful completion of school and development of social ability (Yang et al., 2022). Therefore, it is very necessary to carry out physical education in middle schools to stimulate the potential of the educated, so as to improve their ability to deal with various negative events encountered in their studies and improve their psychological resilience (Gary et al., 1997). Perry and Weinstein (1998) puts forward the model of school adjustment theory, from the perspective of ecology research early school adjustment of students, put forward some study motive structure on school adjustment is of great significance to the, such as learning motivation of success and failure attribution, goal orientation, the ability of cognition, to be able to enhance the degree of the persistence of the students in learning. In the field of academic adaptation, it is suggested that good academic adaptation is not only about having relevant skills, but also about maintaining a stable level of motivation. Learning motivation is the most important aspect of learning adaptation, the driving factor of learning, and the internal reason and motivation for causing and maintaining learning (Pintrich, 2003). In the physical education teaching, teachers should cultivate and stimulate the students’ learning motivation through physical exercise, make full use of the physiological and psychological characteristics of middle school students, and carry out the correct ideal and belief education. Based on the internalization mechanism of self-determination theory, it is concluded through experimental research that stimulating students’ interest in physical education, clarifying the goal, promoting the internalization of emotion, and building a motivational atmosphere are of great significance for improving students’ motivation to participate in physical education learning and improving the effect of physical education teaching (Berki and Tarjányi, 2022).

### Physical exercise positively predicted the chain mediator of junior high school students’ school adaptation

Psychological resilience has a significant positive correlation with sports learning motivation, which is consistent with the research results of Zhao and Chen (2018) and Peng (2013). As an important component of mental resilience, support, and personal strength can promote individuals to effectively cope with stress. The cognitive evaluation theory points out that competency perception can increase the internal motivation of individuals. When the level of support and personal strength reaches a higher level, they can perceive a higher level of learning competence, and then improve the internal learning motivation. Focusing on the mechanism of physical exercise–psychological resilience–sports learning motivation–school adaptation to this path, physical exercise can improve the psychological resilience of junior high school students. The improvement of psychological resilience is accompanied by the improvement of sports learning motivation. On this basis, the positive self-awareness combined with good emotional experience can improve the school adaptation ability of junior high school students. This is consistent with the protective factor-protective factor model that one protective factor can strengthen the effect of another protective factor, that is, psychological resilience, as a protective factor for individual mental health, can strengthen the effect of sports learning motivation, which is also a protective factor. Results show that the psychological resilience to physical education learning motivation in the study of the chain of intermediary effect is feasible, in the physical training with the school to adapt to the relationship between the partial intermediary role, therefore, in thinking about junior middle school students physical exercise and the relationship between school adjustment, to pay attention to psychological resilience and physical education learning motivation plays an important “bridge” role.

### Research deficiencies and future prospects

Although this study has explored the internal mechanism of physical exercise affecting junior high school students' school adaptation, since this study adopted self-reported report, there are the following problems: (1) All the subjects in this study are junior high school students, and subsequent studies can investigate other groups of adolescents to expand the sample representation; (2) This study is a correlation study in nature and cannot prove causality. Longitudinal tracking of the relationship between experimental intervention variables should be adopted in the future to improve the external validity of the study. (3) This study only analyzed the mediating effect of the total scores of physical exercise, psychological resilience, sports learning motivation and school adaptation, and the relevant effects of each dimension need to be further studied.

## Conclusion

Physical exercise can significantly positively predict the psychological resilience and sports learning motivation of junior high school students, which is of great significance to the improvement of psychological resilience and the stimulation of sports learning motivation of teenagers. Physical exercise can directly improve the school adaptation of junior high school students, and it can indirectly affect the school adaptation of junior high school students through psychological resilience or sports learning motivation, and indirectly affect the school adaptation through the chain intermediary effect of psychological resilience and sports learning motivation. There are three mediating pathways in this model: (1) mediating pathways through psychological resilience; (2) Through the mediating path of sports learning motivation; (3) through the psychological resilience and sports learning motivation of the chain intermediary path.

## Data availability statement

The original contributions presented in the study are included in the article/Supplementary material, further inquiries can be directed to the corresponding authors.

## Author contributions

M-ZB and S-JY designed the study, collected, and analyzed the data, and wrote the manuscript. Q-SM, X-LW, CL, and K-LG revised the manuscript. All authors contributed to the article and approved the submitted version.

## Conflict of interest

The authors declare that the research was conducted in the absence of any commercial or financial relationships that could be construed as a potential conflict of interest.

## Publisher’s note

All claims expressed in this article are solely those of the authors and do not necessarily represent those of their affiliated organizations, or those of the publisher, the editors and the reviewers. Any product that may be evaluated in this article, or claim that may be made by its manufacturer, is not guaranteed or endorsed by the publisher.
